# Systematic Review into Diagnostics for Post-Kala-Azar Dermal Leishmaniasis (PKDL)

**DOI:** 10.1155/2013/150746

**Published:** 2013-07-09

**Authors:** Emily R. Adams, Inge Versteeg, Mariska M. G. Leeflang

**Affiliations:** ^1^Liverpool School of Tropical Medicine, Pembroke Place, Liverpool L3 5QA, UK; ^2^Royal Tropical Institute, KIT Biomedical Research, 1105 AZ Amsterdam, The Netherlands; ^3^Department of Clinical Epidemiology, Biostatistics and Bioinformatics, Academic Medical Centre, Meibergdreef 9, 1105 AZ Amsterdam, The Netherlands

## Abstract

Identification of post-kala-azar dermal leishmaniasis (PKDL) is important due to the long and toxic treatment and the fact that PKDL patients may serve as a reservoir for visceral leishmaniasis (VL). We summarized the published literature about the accuracy of diagnostic tests for PKDL. We searched Medline for eligible studies investigating the diagnostic accuracy of any test for PKDL. Study quality was assessed using QUADAS-2. Data were extracted from 21 articles including 43 separate studies. Twenty-seven studies evaluated serological tests (rK39 dipstick, ELISA, DAT, and leishmanin tests), six studies molecular tests, eight microscopy, and two cultures. Only a few of these studies reported a valid estimate of diagnostic accuracy, as most were case-control designs or used a reference standard with low sensitivity. The included studies were very heterogeneous, for example, due to a large variety of reference standards used. Hence, no summary estimates of sensitivity or specificity could be made. We recommend well-designed diagnostic accuracy trials that evaluate, side-by-side, all currently available diagnostics, including clinical symptoms, serological, antigen, molecular, and parasitological tests and possible use of statistical modelling to evaluate diagnostics when there is no suitable gold standard.

## 1. Introduction

Post-kala-azar dermal leishmaniasis (PKDL) is a skin disorder that often appears after treatment for visceral leishmaniasis (VL) patients. It has also been reported in individuals without prior history of VL as well as those undergoing treatment for VL. The protozoan parasite *Leishmania donovani* is the only causative agent. Clinical manifestations of PKDL are macular, maculopapular, and nodular rash in people who are otherwise well and recovering, although more serious manifestations of facial ulcers can occur. In Sudan approximately 50% of VL patients go on to develop PKDL [1]; but in the Indian subcontinent only 5–10% develop this infection [2]. The clinical presentation of PKDL varies, making diagnosis difficult, especially in those without a history of VL.

Accurate diagnosis of PKDL is important due to the long and toxic treatment with antileishmanial drugs. Treatment can last up to 6 months, and the drugs can have serious side-effects for patients and is a waste of medical and economic resources. Therefore, a testing regime with a high specificity is essential to avoid false positive results and hence to avoid waste and unnecessary treatment of patients with toxic drugs. On the other hand, diagnostics for PKDL should also have a sufficiently high sensitivity as PKDL patients may be a risk of continuing transmission of VL. 

The first step in diagnosis is assessing clinical signs and symptoms, and in some areas cases are diagnosed on clinical symptoms alone [1]. However, there is a large geographical variation in clinical presentation, especially between East African and the Indian subcontinent, and PKDL can be confused with other skin disorders [2]. Recently, pictorial training aids have been created as a guide for health workers to distinguish PKDL patients from other skin disorders [3]. 

In other areas, clinical signs and symptoms along with history of VL, living in an endemic area, and positive antibody tests are used to diagnose patients, without further confirmation of disease. 

Both approaches are problematic. Clinical signs and symptoms may not be accurate enough, and diagnosis depends on the training of an individual clinician. In addition, around 10% of cases have no history of VL, and 10% of cases have no positive serological test [2]. Many patients post-VL treatments have a positive antibody test as the antibodies are known to stay in the body for some years making a strict clinical definition important. This may hamper the specificity of these tests.

Confirmation of diagnosis is usually done by skin slit smear (SSS) microscopy or histopathology. However, the reported sensitivity of SSS microscopy is, at best, 40–60% from patients with nodular lesions [2, 4] and even lower in patients with macular lesions. In addition, parasite load between different clinical presentations may differ, which may mean that some diagnostic tests are more suitable for some patients than others [4]. The advantage of microscopy is the acknowledged high specificity, which leads to low numbers of patients unnecessarily treated with antileishmanial drugs. However, because of the woeful sensitivity many centres do not use this test. 

The aim of this overview is to systematically collect and summarize all of the published literature about the accuracy of diagnostic tests for PKDL. This involves the assessment of the quality of these studies and extracting the diagnostic accuracy data. In the discussion section, we will highlight the implications of our findings.

## 2. Material and Methods

### 2.1. Literature Search

A search was performed in Medline, through PubMED to find all the literature associated with PDKL and diagnostics. The following search terms were used: post-kala-azar, dermal leishmaniasis, PKDL, post-AND visceral leishmaniasis (see the appendix). This broad search enabled the identification of all PKDL articles that might involve PKDL diagnostics. The title and abstract of all identified papers were screened in order to exclude papers not involved in diagnosis of PKDL. The full text articles were read for inclusion in the systematic review.

### 2.2. Inclusion Criteria

Articles were included in the systematic review if they investigated the diagnostic accuracy of any test for PKDL. This is best done in a study including a consecutive series of eligible patients who will all undergo the test under evaluation (the index test). Then a reference standard, or gold standard, should be applied to all patients, enabling verification of the index test results. The reference standard defines whether someone has the target condition or not; studies were not included if they did not use a reference test or confirmation method. Such a design is typically a cross-sectional design, but because we suspected that there would not be many of these studies available, cohort designs and case-control type designs were also included. Case control designs select patients who have the target condition at the start of the study (to calculate sensitivity), and they select a separate control group of people who do not have the target condition (to calculate specificity). If a mixture of control groups was used, then the most appropriate was chosen, that is, European controls and healthy endemic controls were omitted if possible, as these are least like the original population in which these tests will be used in practice.

Studies were included if they used a reference standard; here this consisted of clinical symptoms plus parasitological diagnosis (e.g., skin-slit smear or other microscopy) or response to treatment. Studies needed to report at least an estimate of sensitivity, and preferably also specificity, or the ability to calculate these from the results.

Any index test was eligible; we expected to find information on serological, molecular diagnostics, and different types of microscopy. We included studies from any patient population.

Articles were excluded if an index test was presented without reference to a gold standard diagnostic, if only clinical symptoms were used as the gold standard article and if a case study presented under 5 patients. 

### 2.3. Data Extraction

Where possible, data for 2 × 2 tables for each index test were extracted. Geographic origin of patients, sample type, study design, control group (if appropriate), and molecular target (if appropriate) were recorded. To assess the risk of bias in the included studies, we used the QUADAS-2 checklist for each study (*n* = 41) [5]. Study selection and performing data extraction were performed independently by authors IV and EA. In the case of disagreements a third author ML acted as a moderator. 

The QUADAS-2 checklist assesses the risk of bias and concerns regarding applicability over four domains: patient selection, index test, reference standard, and flow and timing. Patient selection was regarded to be at high risk of bias if a case-control design was used; cases may have gone through confirmation methods before they were regarded real cases, making it easier for an index test to detect a “case.” The controls groups were often healthy controls, or even healthy European controls. They are at the other end of the disease spectrum, showing no signs of leishmania and therefore more likely for any test to become negative. This leads to overestimation of sensitivity and specificity in these studies. 

The reference standard was regarded to be at high risk of bias if a nonoptimal reference standard was used, for example, any kind of microscopy, or if the assessors interpreting the reference standard were not blinded for the results of the index test.

Flow and timing was regarded to be at high risk of bias when there was too much time between the index test and the reference standard, when not all patients received the (same) reference standard, or when not all enrolled patients ended up in the 2 × 2 tables.

Concerns regarding applicability have to do with the representativeness of the studies. As case-control studies do not reflect the actual variation in patients, these were all scored “high” concern regarding applicability of the patient population. The index test was thought to be of high concern regarding applicability if a test used in-house ELISAs or PCRs, due to no standardization. 

Any of the above items were scored “unclear” if the study did not report on the item, or if it was not clear whether high or low risk or concern would have been appropriate. 

### 2.4. Data Analysis

Data were entered into RevMan (version 5.2) [6] to create forest and ROC plots of sensitivity and specificity per study. Meta-analysis was considered if at least four studies per test were found that did not include healthy (endemic) controls; however, this criterion was not met for any of the tests included in the review.

## 3. Results

### 3.1. Included Studies

**Figure 1:**
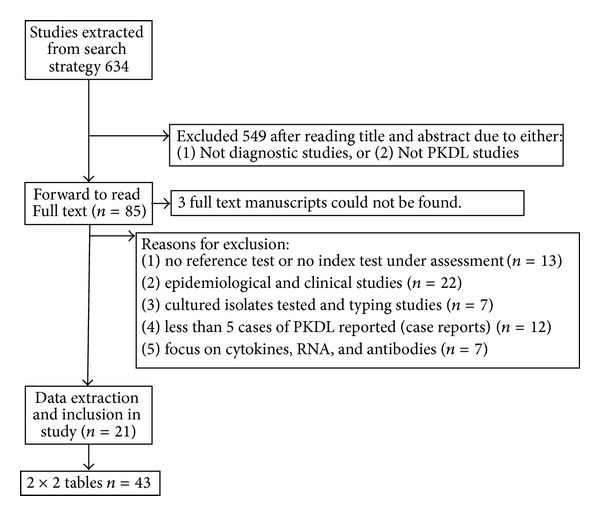
Flow of included studies.

The title and abstract of 634 studies were read and taken forward to read the full text if the inclusion criteria were likely to be fulfilled (see Figure 1). Eighty-five full text articles were read, and data extraction was possible from 21 articles [4, 7–26] which included 43 separate 2 × 2 tables (hereafter referred to as studies). Of three articles, the full text could not be retrieved, either from the library, online or by emailing corresponding authors. 

We included 21 articles, containing a total of 635 people with PKDL and 468 people without PKDL. Patients from 17 articles came from the Indian subcontinent (India, Bangladesh, or Nepal). Four articles came from Sudan. Most articles reported the results for only one index test or one sample type, but some reported up to 5 2 × 2 tables for other index tests. These multiple 2 × 2 tables overlapped in included patients and controls. Twenty-seven studies concerned serological tests (rK39 dipstick or ELISA = 10; DAT = 5; leishmanin skin test = 2, and 10 other serological tests), six were on PCR, four on immunohistochemical staining, one on hematoxylin and eosin histochemical staining, three on microscopy of skin-slit smears, two on culture, and one on histological sections. See Figures 3 and 4 for the ROC and forest plots of included studies. 

We did not formally test for heterogeneity, but based on the variation in included index tests, reference standards, and study designs, we concluded that the heterogeneity in the 43 studies was large. There are 6 types of index test of which the serological test contained several different antigens (rK39, DAT, and unnamed antigens) for antibody detection and several different readouts including Lateral Flow Devices (LFD), ELISA, and agglutination; the molecular tests used several different target genes, protocols (standard versus nested), and different read-out systems (realtime versus electrophoresis). There were 5 different types of references standard and many types of control group for the case/control studies. All of these parameters meant that pooling for statistical analysis was not possible in this group of studies.

### 3.2. Methodological Quality

The main problem in terms of risk of bias is in the patient selection (see Figure 4); this is due to the large proportion of case series and case-control studies [27]. Only six 2 × 2 tables were based on a cross-sectional study, while 28 studies were based on a case-control design. Control groups ranged from healthy volunteers from Europe, healthy endemic controls, and patients with diseases in the differential diagnosis of PKDL, for example, leprosy. Our dataset also contained nine cases series (positive cases only), for which we could only calculate the sensitivity and not specificity. 

Another problem in the included studies was the reference standard. All included studies used clinical symptoms as part of the reference standard, combined with microscopy of skin-slit smears (10 out of 22 articles), response to treatment (6 articles), microscopy with hematoxylin and eosine staining (2 articles), or histopathology or microscopy of a variety tissues or aspirates. These tests have a low sensitivity and therefore high likelihood of false negative results by the index test. 

Almost all studies (42 studies) scored as “unclear” on the timing and flow section, due to lack of reporting; one article scored high as patients were selected from a large group of samples, but the reasons for this were not explained [16].

### 3.3. Serological Tests

The largest group of studies evaluated a serological test. Eight studies evaluated the rK39 lateral flow devices. Their sensitivity ranged from 91% to 100%, while the specificity ranged from 0% to 100%. The three consecutive studies were very variable in their estimates for specificity (0%, 8%, and 89% resp.) and reported 100% sensitivity. 

Five of them assessed the Direct Agglutination Test (DAT), the sensitivity of the case-control studies ranged between 94% and 100%, while the specificity was 100% in four studies and 40% in one study. 

Ten case-control studies evaluated other serological tests (Figure 3); sensitivity was estimated between 67–100% and specificity 81–100%; again this is likely to be an overestimation due to the study design.

### 3.4. Molecular Tests

Six studies evaluated the diagnostic accuracy of molecular tools for PKDL on skin biopsies or skin slit smears [8, 11, 22]. Their sensitivities are high, ranging from 94% to 100%, but their specificities are variable, ranging from 8% to 100%. Low specificity is especially apparent in the 2 studies with a consecutive design Nasreen et al. [8] 8% and Osman et al. [22] 23–25%. There are 2 molecular studies which use response to treatment as a reference standard; here the sensitivity ranges between 94–96% and the specificity 90–100%. 

### 3.5. Other Tests

**Figure 2:**
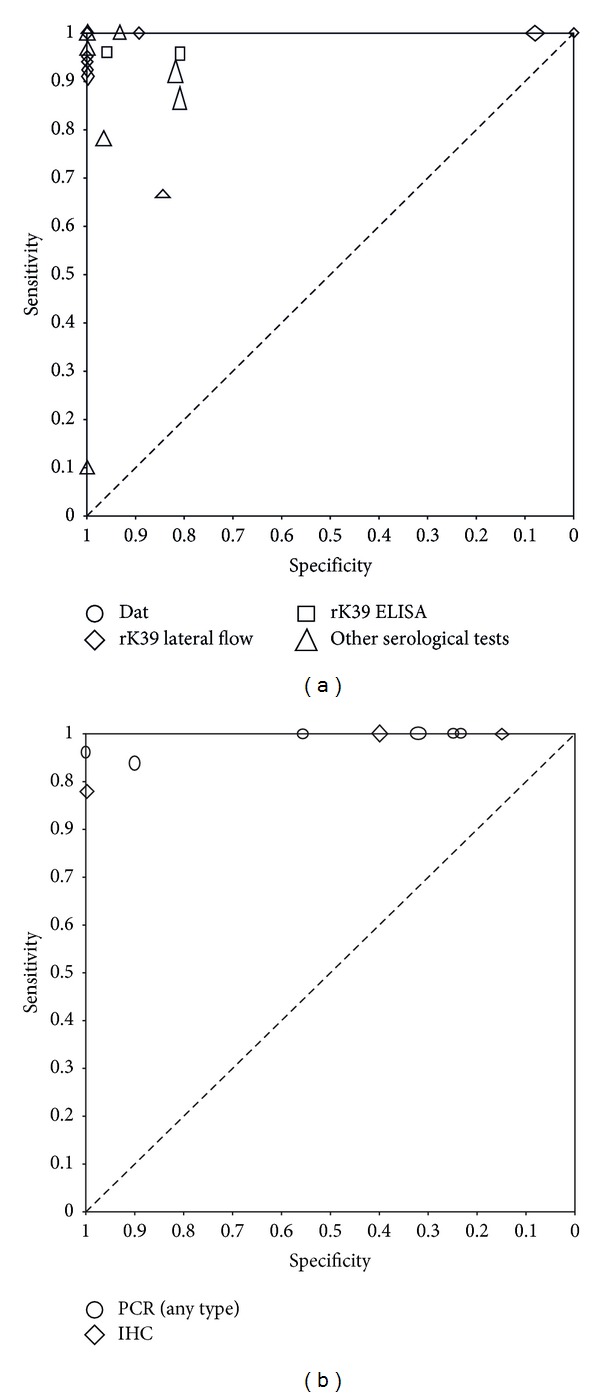
(a) Raw ROC plot of the serological tests. Every symbol refers to the sensitivity (*y*-axis) and specificity (*x*-axis) of a test in a study. The height of the symbol represents the number of diseased in the study, and the width of the symbol represents the number of nondiseased in the study. Circles = DAT; diamonds = rK39 LF; squares = rK39 ELISA; triangles = other serological tests. The majority of studies are clustered into the upper hand left corner, indicating a near perfect sensitivity and specificity; however, poor patient selection must be taken into account when drawing conclusions. (b) Raw ROC plot of PCR and IHC. Every symbol refers to the sensitivity (*y*-axis) and specificity (*x*-axis) of a test in a study. The height of the symbol represents the number of diseased in the study, and the width of the symbol represents the number of nondiseased in the study. Circles = PCR; diamonds = IHC. The majority of studies have a sensitivity of above 90%, but specificity varies widely.

**Figure 3:**
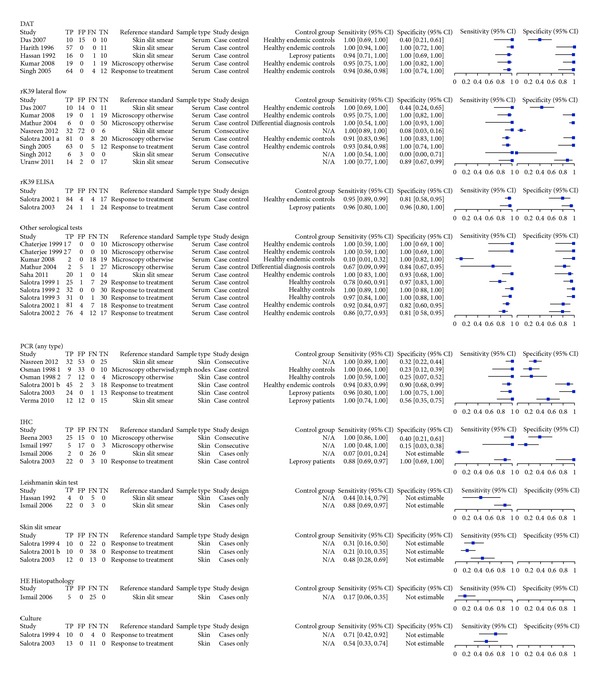
Forest plots of included studies. Overview of all 2 × 2 tables with forest plot (TP = true positives; FP = false positives; FN = false negatives; TN = true negatives; DAT = direct agglutination test; PCR = polymerase chain reaction; IHC = immunohistochemistry). Numbers 1–4 refer to different set of data from the same paper.

**Figure 4:**
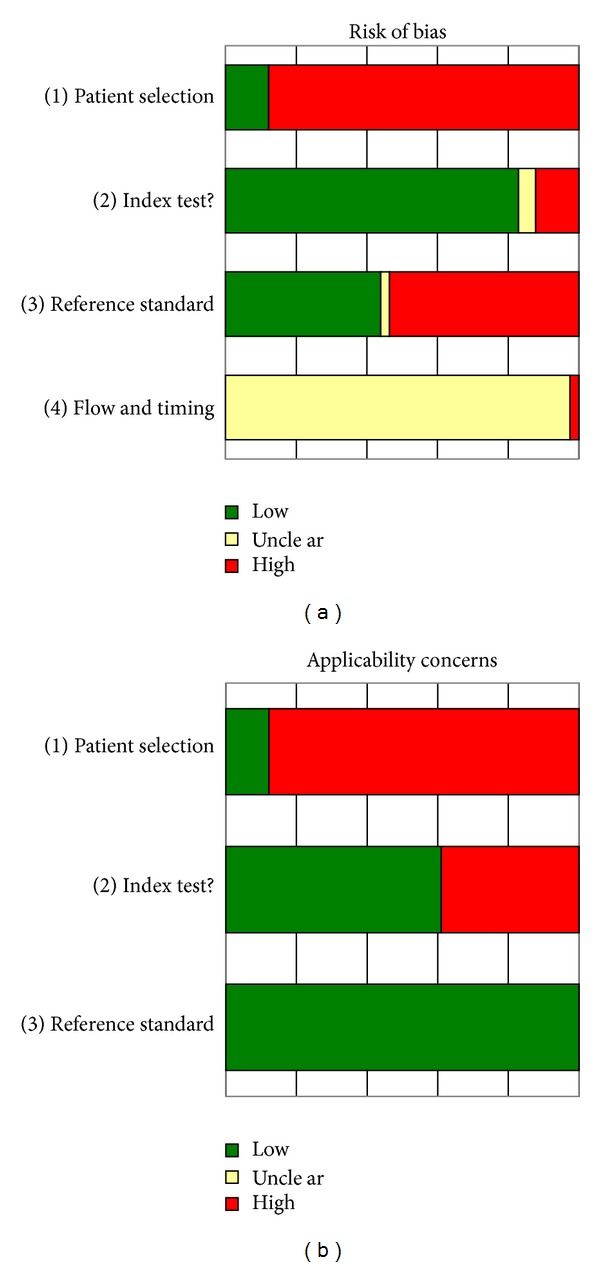
QUADAS-2 results. (a) shows the risk of bias from each of the 41 2 × 2 contingency tables. Note that less than 20% of the included 2 × 2 tables had a low risk of bias for patient selection. (b) shows the risk of applicability of the patients, index, and reference tests to use in PKDL areas.

Leishmanin skin test, histopathology, microscopy of SSS, and IHC were also assessed in several studies. The results can be found in Figure 2. Of these, only for the IHC both sensitivity and specificity were reported. The other tests were assessed using case series.

## 4. Discussion

Studies evaluating the diagnostic accuracy of tests for PKDL are insufficiently rigorous to draw firm conclusions with regard to their sensitivity and specificity. The main problem is in the selection of patients, as most studies we retrieved were case-controls designs, including healthy (endemic) controls. The other problem these studies face is the lack of a reference standard that is able to define beyond reasonable doubt whether a patient has PKDL or not. There are few well-designed (consecutive) diagnostic accuracy studies for PKDL, those that were found use microscopy as a reference standard. Microscopy is shown to have a low sensitivity, as seen here in three studies [17, 19, 20] where microscopy was evaluated against response to treatment as a reference standard. A reference standard with a low sensitivity may cause many false positive reactions in the index tests, thereby producing a low specificity; consequently assessing the index test for accuracy becomes extremely difficult. This is seen in most of the consecutive studies we included, where we found a wide range of variation in specificity. 

Some authors have managed to get round the lack of a gold standard by using the response to treatment as a reference standard. Although the use of anti-leishmanial drugs should not affect other infections, administering a toxic drug to patients who do not really have PKDL is not advisable. It will therefore be difficult to combine the reference standard of treatment response with a neat consecutive study design. 

In studies using response to treatment as a gold standard, all cases (suspected patients) appear to respond positively to treatment. This may suggest that (i) clinical suspicion alone maybe a good diagnostic tool in itself, (ii) that patients can also self-heal over time, or (iii) that the treatment has an effect on more than one pathogen. In order to test this hypothesis, clinical diagnosis could be treated as an index test in a cohort of PKDL suspected patients. All other diagnostics including serological, molecular, and parasitological tools could then act as the reference test for which to compare a clinical diagnosis. In practice, a clinical diagnosis is frequently used for PKDL, especially in Sudan; confirmation of the efficacy of this diagnostic would be extremely beneficial. We found no studies evaluating clinical signs and symptoms as an index test.

Although the serological tests seem to have reasonably high (>80%) specificity, one would expect lower specificities under field circumstances, as it is known that antibodies can stay in the body for a long period of time, up to many years. The low specificity in some of the molecular test evaluations may have a different cause; the sensitivity of all PCR tests, regardless of target gene, was high. Due to the imperfectness of microscopy as a gold standard and the high sensitivity of the PCR tests, a (truly) positive test result by PCR may coincide with an infected patient in whom microscopy missed the diagnosis, thus ending in a false negative results. 

Several types of microscopy are reported, including light microscopy of SSS, histopathology, haematoxylin and eosin histopathology stain, and immunohistochemistry. Due to the lack of studies it has not been possible here to assess the differences in sensitivity between these tests. However, it is likely that there is a wide range in diagnostic accuracy both in different microscopy tests and the type of tissue and patient (macular versus nodular). Future assessment of microscopic tools should also remain a priority due to the wide use of this diagnostic. 

 Of the 21 reported articles, only 4 studies were done in East Africa. Due to known differences in performance of other diagnostics, including the rapid diagnostic tests for VL [28], it is important that new diagnostics are also assessed in both the ISC and East Africa. Several East African articles were excluded from this review as PKDL was not positively diagnosed (i.e., no gold standard); this becomes extremely important in diagnostic evaluations where the aim is to evaluate index tests in comparison to the reference tests.

Limitations to this systematic review include our search and selection process. Although we feel that our broad search strategy should have retrieved all relevant articles, there were a few articles to which there was no access. Another, and perhaps more relevant, limitation is the possibility of publication bias, whereby poor results of diagnostics are not published. As most of our included studies report very high sensitivities and specificities, the likelihood of publication bias should certainly be considered, although it is however not possible to assess the impact of this, as testing for publication bias in diagnostic test accuracy studies has its own limitations and because prepublication of study protocols in this field is not encouraged. 

Due to poor reporting of study characteristics in the included studies it was difficult to assess the actual merit of the study results. No additional information about the flow and timing of study methods and test reading was reported, like information on blinding and random assortment of samples. This may particularly be a problem for the evaluation of molecular tests. Due to the high sensitivity of molecular tests, controls must be taken along through the processes of sampling, DNA extraction, and amplification with appropriate timing (i.e., in an assortment of positive samples) to ensure no contamination or bias. 

## 5. Conclusion

Although there are several potentially useful diagnostic tests for PKDL, there are few studies of high quality mostly due to the poor reference standard of clinical symptoms plus microscopy. This makes it difficult to assess new index diagnostics since microscopy is so poorly sensitive and clinical symptoms are not specific. Very few of the studies have included consecutively enrolled patients for the assessment of diagnostic tools. This leads to an artificial increase in the diagnostic accuracy of tests as only known positives and negatives are tested; more recently several consecutively designed studies have been published, and we encourage the move to this study design which assesses index tests against a whole range of patients and leads to less bias in results. 

We recommend well-designed diagnostic accuracy trials that evaluate, side-by-side, all currently available diagnostics for PKDL. This would include clinical symptoms and serological, antigen, molecular, and parasitological tests. A future development may be the use of statistical modeling to evaluate diagnostics [29] when there is no suitable gold standard to refer to as is the case with PKDL; this would also allow clinical diagnosis to be assessed as an index test.

The aim of diagnostic evaluations should not be to find a test with a perfect sensitivity and specificity, but to get a valid estimate of its value in health practice. Therefore, as a community, we must prioritise valid evaluations of all available diagnostics in well-designed diagnostic accuracy studies.

## Appendix

Search items are as follows: post kala-azar [tiab] OR dermal leishmaniasis [tiab] OR PKDL [tiab] OR post[All Fields] AND (“leishmaniasis, visceral”[MeSH Terms] OR (“leishmaniasis”[All Fields] AND “visceral”[All Fields]) OR “visceral leishmaniasis”[All Fields] OR (“kala”[All Fields] AND “azar”[All Fields]) OR “kala azar”[All Fields]).
